# The IL-23/-17 Axis in PsA: What Have We Learned in the Last 20 Years?

**DOI:** 10.31138/mjr.140826.psv

**Published:** 2026-09-01

**Authors:** Fotini Theodoridou, Olga Katsouli, George E. Fragoulis

**Affiliations:** 1 First Department of Propaedeutic and Internal Medicine, National and Kapodistrian University of Greece, Athens, Greece;; 2 School of Infection and Immunity, University of Glasgow, Glasgow, United Kingdom

**Keywords:** psoriatic arthritis, IL-17, IL-23

Psoriatic arthritis (PsA) is a chronic, immune-mediated arthropathy affecting about 20–30% of patients with psoriasis.^1^ Clinical features include arthritis, enthesitis/dactylitis, axial involvement, and skin manifestations (psoriasis and nail involvement). Apart from these common hallmarks, people living with PsA are also at higher risk for extra-musculoskeletal manifestations such as inflammatory bowel disease and uveitis. Furthermore, comorbidities mainly belonging in the cardiometabolic spectrum and mental health disorders, are encountered more frequently in PsA.^2^ Although the underlying pathogenetic mechanisms of PsA are yet to be completely elucidated, research indicates complex pathways that take genetic predisposition, e.g. certain HLA alleles,^3^ metabolic and environmental factors into account. As such, genetic variants regarding immune response to gut dysbiosis, coupled with mechanical stress, obesity and smoking, trigger an exaggerated inflammatory response to the affected tissues,^4^ resulting in cytokine pathway activation and autoimmune/autoinflammatory phenomena.

Over the last 20 years, significant advancements have been made, and data have been accumulated showing the fundamental role of IL-23 and IL-17 in the PsA pathogenesis,^5,6^ reflected also in the development of monoclonal antibodies targeting these molecules. Indeed, nowadays we have more than 6 drugs in this therapeutic category (namely: secukinumab, ixekizumab, bimekizumab, ustekinumab, risankizumab, guselkumab, tildrakizumab) while more are in the pipeline.^7^

## IL-23/IL-17 AND THEIR ROLE IN PSA PATHOGENESIS

IL-23 is a cytokine of the IL-12 family composed of two subunits. It is mainly produced by dendritic cells and macrophages in response to inflammation signalling driven by bacterial and fungal infections.^8^ Its main role is the induction of αβ T CD4+ cell differentiation into T helper type 17 cells (Th17).^9^ By binding to its corresponding receptor, IL-23R, expressed by the naïve CD4+ T cells, it promotes the upregulation of retinoid-related orphan receptor gamma transcription factor (RORγt) and the subsequent expression of IL-17 by Th17 cells, in a pathway that is often described as the IL-23/IL-17 axis.^10^ This axis is thought to play a central role in chronic inflammatory disorders such as ankylosing spondylitis, inflammatory bowel disease, psoriasis and PsA.^11^

IL-17 is a large cytokine family that comprises six cytokines, from IL-17A to IL-17F. Despite Th17 cells being the main source of IL-17, several studies have noted that it is also being produced by other cell populations. Research points to mucosal-associated invariant T cells (MAIT cells), invariant natural killer cells (iNKT), innate lymphoid cell group 3 (ILC3) and γδ T cells. These cell groups have been found to express both IL-23R and RORγt and can produce IL-17 irrespective of IL-23 production.^12,13^

## THEORIES FOR PsA PATHOGENESIS

Given the complex nature of PsA and the diverse clinical manifestations that may have, different theories have been suggested to underlie the pathogenesis of PsA.

**Figure 1.**
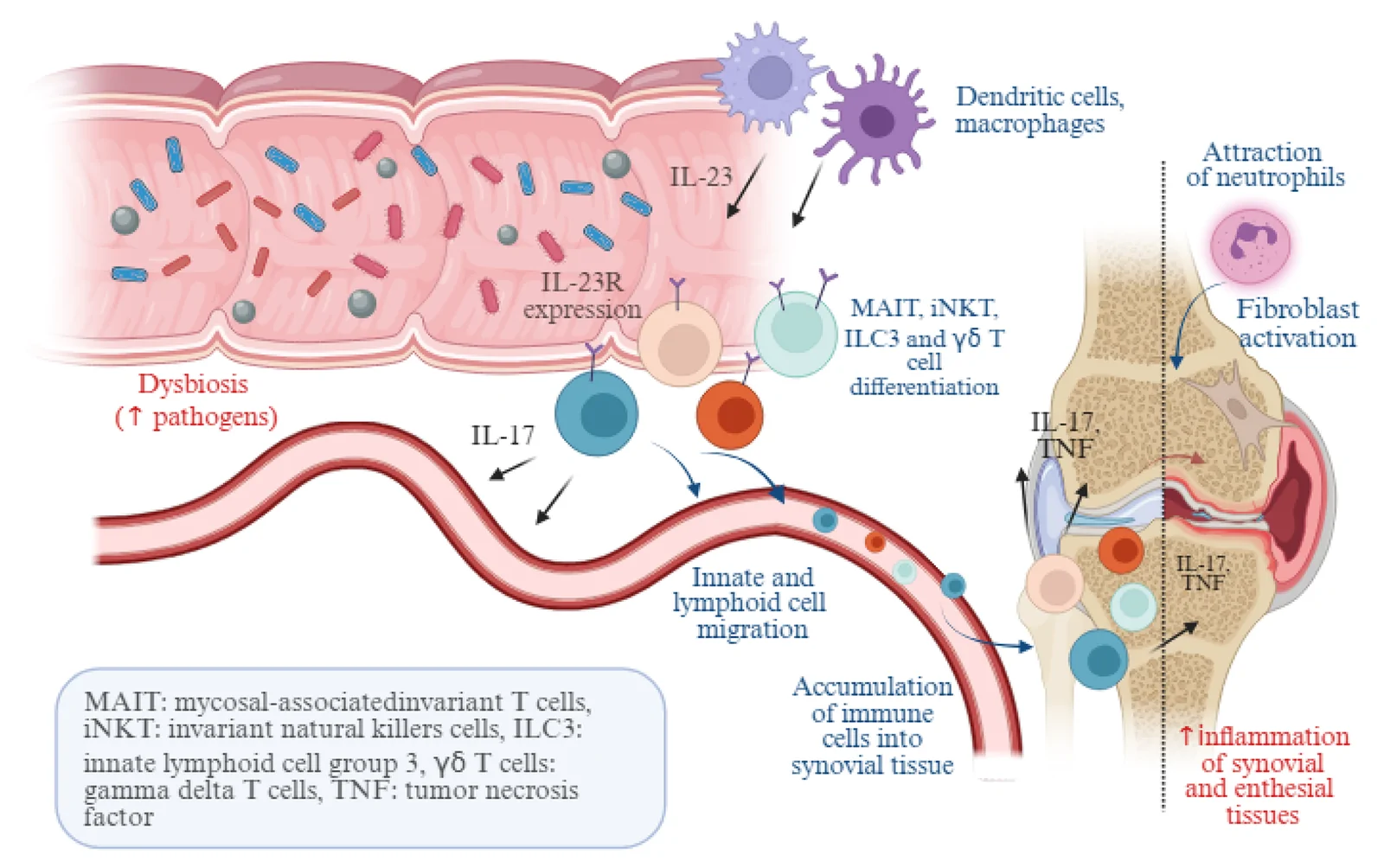
The gut-joint axis. Cells and cytokines involved.

Some investigators have suggested that the “first hit” could be in the bowel. The disruption of gut microbiota, also known as dysbiosis, affects immune and metabolic homeostasis (**Figure 1**). Subsequently, IL-23-producing cells are activated in the intestinal lamina, which in turn set off the differentiation of MAIT, ILC3 and γδ T cells. Accompanied by pro-inflammatory cytokine release, the activated cells pass through the permeable gut barrier and migrate to the musculoskeletal components of the affected individual through the bloodstream.^4^ Future studies of this so-called gut-joint axis could potentially pave the way for the approval of microbiome-targeting therapeutics^14^ or even faecal transplant therapy^15^ for PsA.

Migration of these cells is also at the centre of the second theory, in which certain T cell populations and myeloid progenitors are thought to migrate from the skin to the joints. As described in the gut-joint theory, IL-23 production from dendritic cells in psoriatic plaques sets off the activation and migration of Th17 cells, cytotoxic T lymphocyte (Tc17) -like CD8^+^ tissue-resident memory T (TRM) cells or ex-resident TRM cells and CD2^+^ M-HCII^+^CD200R^+^ myeloid cells. Adhesion of these cells to musculoskeletal compartments promotes synovial and entheseal inflammation, making psoriatic skin an effective “immunological reservoir”.^16^

The third theory places the entheseal niche in the centre. The surge of immune cells in synovial tissue, particularly at the entheses, evokes an inflammatory response that causes a classic clinical finding: enthesitis. Current studies suggest that the influx of MAIT, ILC3, iNKT, and γδ T cells, facilitated by the release of cytokines such as IL-17 and tumour necrosis factor α (TNF-α), further amplifies the infiltration of peripheral immune cells and stimulates tissue fibroblasts. Additionally, stimulation of tenocytes due to mechanical overload leads to the release of chemokines such as CCL20, promoting T cell recruitment and Th17 attraction on ligament and tendon cites.^17,18^

These crucial events, coupled with physiological stress and trauma.^19^ are pivotal steps in the ligament/tendon inflammation, bone remodelling, and deep scarring of the enthesitis pathogenesis.^20^ People with PsA have a lower threshold for the development of the so-called “deep” Koebner effect, analogous to the cutaneous manifestation of psoriatic disease.^21^ Meanwhile, recent evidence suggests that enthesitis might be the main precursor for the development of dactylitis, or inflammation of the entire digit.^22^

## CLINICAL EVIDENCE FOR IL-23/-17

Targeting both IL-23 and IL-17 results in significant improvements across multiple PsA domains, as shown by phase III trials and real-world data.

The first clinical evidence came from the pivotal PSUMMIT-1 and PSUMMIT-2 trials of ustekinumab, which demonstrated that targeting the shared IL-12/IL-23 p40 subunit results in meaningful improvements in peripheral arthritis and psoriasis as well as in other features of PsA, like enthesitis and dactylitis.^23,24^ Subsequently, selective IL-17A inhibition with secukinumab and ixekizumab also proved to be efficacious in all musculoskeletal manifestations of PsA, confirming IL-17 as a key effector cytokine in PsA.^25–27^ In the SPIRIT-H2H trial, ixekizumab was superior to adalimumab when used without concomitant MTX in the combined ACR50 and PASI100 endpoint (p=0.002). Additionally, more patients achieved MDA (p=0.016) and VLDA (p=0.037) after 52 weeks.^28^ Moreover, the MAXIMISE trial is currently the only RCT showing the efficacy of secukinumab in symptoms and signs of axial PsA through week 52.^29^ Selective IL-23p19 inhibitors further expanded our therapeutic armamentarium, with guselkumab and risankizumab demonstrating robust and sustained efficacy across multiple disease manifestations in the phase III DISCOVER^30,31^ and KEEPsAKE trials,^32,33^ respectively. Apart from their clinical effectiveness, evidence supports their role in long-term structural disease control in patients with active PsA, as shown in the phase 3b APEX trial^34^ and in the recently presented five-year data from the phase 3 KEEPsAKE 1 trial of Risankizumab.^35^

Finally, the advent of the dual IL-17A/F inhibitor, bimekizumab, has revolutionised our treatment strategies. In the BE OPTIMAL trial, it was superior to placebo for its primary endpoint, showing an ACR50 of 44% vs 10% at week 16 (p<0.001) and also demonstrated better results in psoriasis, enthesitis, dactylitis and radiographic progression in bDMARD-naïve patients.^36^ The BE COMPLETE trial confirmed this efficacy in previously TNFi-experienced patients, with the magnitude of effect being comparable to that seen in bDMARD-naïve patients.^37^ Importantly, according to the late-breaking results of the phase 3b BE BOLD study, bimekizumab was superior to risankizumab for the primary endpoint of ACR50 at week 16 (49.1% vs 38.4%, p=0.0078), maintaining a comparable safety profile, despite a higher incidence of mild-to-moderate Candida infections. Although the first secondary endpoint of MDA at week 16 did not reach statistical significance, likely due to the study’s design, BE BOLD represents the first direct comparison of IL-17 and IL-23 inhibition strategies in PsA.^38^

The efficacy and safety of most of the above treatments targeting the IL-23/IL-17 axis have been well established in everyday clinical practice. Real-world data demonstrate sustained improvements in clinical outcomes, acceptable drug persistence rates and a favourable safety profile for inhibitors of the IL-23/IL-17 axis across different populations, including those with multiple comorbidities, long-standing or difficult-to-treat disease.^39–43^ Among these, IL-17 inhibitors have the strongest real-world evidence. In the multinational SERENA study, secukinumab demonstrated high effectiveness across all musculoskeletal domains in PsA patients, with a 74.9% retention rate over a 2-year period. Two-thirds of patients had prior biologic exposure and 90% of them discontinued previous treatments due to lack of efficacy.^39^ Data derived from the Spanish BIOBADASER registry confirmed the above results both for secukinumab- and ixekizumab-treated, naïve or bDMARD-experienced PsA patients.^40,41^ Although real-world evidence with IL-23 inhibitors is more scarce, data from the CorEvitas PsA registry^42^ and the Italian multicentre registry GISEA [43] further support the multidomain efficacy of guselkumab observed in RCTs, even in difficult, treatment-resistant patients. As physicians’ experience with risankizumab and bimekizumab expands, large observational studies will further determine their position in PsA treatment algorithms.

## POSITION OF THE ANTI-IL-17 AND ANTI-IL-23 IN THE THERAPEUTIC ALGORITHM

Thankfully, treatment options in PsA are not limited only in monoclonal antibodies targeting IL-17 and IL-23; various other therapeutic regimens like TNF-, JAK-and PDE4- inhibitors exist. Overall, treatment selection should be personalised, based on specific disease manifestations and associated comorbidities that guide or preclude the inhibition of each cytokine pathway.^44,45^ Both the 2023 EULAR^44^ and the updated GRAPPA treatment recommendations^45^ for PsA recognise IL-23 and IL-17 inhibitors as potential therapeutic options for patients with prominent peripheral arthritis who have an inadequate response to csDMARDs, at the same level as TNF and JAK inhibitors. For patients with significant skin involvement, both inhibitors are prioritised because of their proven superior efficacy in psoriasis compared with other bDMARDs^44,45^ Regarding axial disease, EULAR recommends IL-17 inhibitors first, based on the MAXIMISE trial’s results, whereas GRAPPA does not discriminate between TNFi and IL-17i.^29,44,45^ Extrapolating the disappointing results of IL-23 inhibition in axial spondylarthritis,^46,47^ the current guidelines do not recommend its use in axial PsA^44,45^ On the other hand, post-hoc analyses and some real-world data suggest that anti-IL-23 could be a potential treatment for patients with axial PsA.^48–50^ Given the emerging understanding of the clinical, anatomical, and immunological distinctions between these two conditions, it is likely that the role of IL-23i will be redefined in future axial PsA treatment guidelines.^51^ Results of the ongoing STAR trial of guselkumab in biologic-naïve PsA patients with MRI-confirmed axial disease are eagerly awaited.^52^ For patients with concurrent inflammatory bowel disease, both recommendations advise avoiding IL-17i due to the risk of IBD exacerbation in patients with active IBD.^53,54^ On the other hand, in patients with recurrent uveitis, monoclonal antibodies against TNF are preferred so far.^44,45^

## FUTURE DIRECTIONS

Understanding the importance of these cytokines in the treatment of PsA, novel drugs targeting these molecules and their respective pathways have been designed and developed, with some already approved in psoriasis. The latest addition to the field was bimekizumab, which targets both IL-17A and IL-17F, in contrast to the IL-17 inhibitors available till now, which target only IL-17A. Of note, new ways of delivering drugs appear to be an alternative strategy. Sonelokimab, a nanobody with three variable heavy-chain-only (VHH) domains that binds IL-17A, IL-17F, and albumin, has already been published with favourable outcomes in phase 2 studies,^7^ while phase 3 trials are underway (NCT06641089). Icotrokinra, an oral IL-23 selective inhibitor (once daily), was recently approved for psoriasis,^55^ while RCTs for PsA are currently underway (NCT06807424, NCT06878404).^56^

In conclusion, although significant progress has been made in the field of PsA treatment over the last 20 years – especially with the appraisal of the role of IL-17 and IL-23 – unmet needs still exist, and disease remission is not being achieved in the long-term for most of our patients. Identification of molecular and clinical endotypes and subsequent treatment stratification are expected to further improve therapeutic outcomes and quality of life.

## References

[1] Alinaghi F Calov M Kristensen LE Gladman DD Coates LC Jullien D Prevalence of psoriatic arthritis in patients with psoriasis: A systematic review and meta-analysis of observational and clinical studies. J Am Acad Dermatol 2019;80:251–265.e19. doi: 10.1016/j.jaad.2018.06.027 29928910

[2] Panagiotopoulos A Fragoulis GE. Comorbidities in Psoriatic Arthritis: A Narrative Review. Clin Ther 2023;45:177–89. doi: 10.1016/j.clinthera.2023.01.006 36737317

[3] Stuart PE Nair RP Tsoi LC Tejasvi T Das S Kang HM Genome-wide Association Analysis of Psoriatic Arthritis and Cutaneous Psoriasis Reveals Differences in Their Genetic Architecture. Am J Hum Genet 2015;97:816–36. doi: 10.1016/j.ajhg.2015.10.019 26626624 PMC4678416

[4] Schett G Rahman P Ritchlin C McInnes IB Elewaut D Scher JU. Psoriatic arthritis from a mechanistic perspective. Nat Rev Rheumatol 2022;18:311–25. doi: 10.1038/s41584-022-00776-6 35513599

[5] Schett G McInnes IB Neurath MF. Reframing Immune-Mediated Inflammatory Diseases through Signature Cytokine Hubs. N Engl J Med 2021;385:628–39. doi: 10.1056/NEJMra1909094 34379924

[6] Fragoulis GE Siebert S McInnes IB. Therapeutic Targeting of IL-17 and IL-23 Cytokines in Immune-Mediated Diseases. Annu Rev Med 2016;67:337–53. doi: 10.1146/annurev-med-051914-021944 26565676

[7] McInnes IB Coates LC Mease PJ Ogdie A Kavanaugh A Eder L Sonelokimab, an IL-17A/IL-17F-inhibiting nanobody for active psoriatic arthritis: a randomized, placebo-controlled phase 2 trial. Nat Med 2025;31:4160–71. doi: 10.1038/s41591-025-03971-6 41053449 PMC12705426

[8] Gaffen SL Jain R Garg AV Cua DJ. The IL-23–IL-17 immune axis: from mechanisms to therapeutic testing. Nat Rev Immunol 2014;14:585–600. doi: 10.1038/nri3707 25145755 PMC4281037

[9] McGeachy MJ Chen Y Tato CM Laurence A Joyce-Shaikh B Blumenschein WM The interleukin 23 receptor is essential for the terminal differentiation of interleukin 17–producing effector T helper cells in vivo. Nat Immunol 2009;10:314–24. doi: 10.1038/ni.1698 19182808 PMC2945605

[10] McKenzie BS Kastelein RA Cua DJ. Understanding the IL-23– IL-17 immune pathway. Trends Immunol 2006;27:17–23. doi: 10.1016/j.it.2005.10.003 16290228

[11] Xynogalas I Karamanakos A Galani A Kougkas N Vassilakis KD Baraliakos X Enthesitis in Spondyloarthritis. From pathogenesis to clinical presentation and imaging: Similarities and differences between PsA and axSpA. Autoimmun Rev 2026;25:104014. doi: 10.1016/j.autrev.2026.104014 41791558

[12] O’Brien-Gore C Gray EH Durham LE Taams LS Kirkham BW. Drivers of Inflammation in Psoriatic Arthritis: the Old and the New. Curr Rheumatol Rep 2021;23:40. doi: 10.1007/s11926-021-01005-x 33909160

[13] Navarro-Compán V Puig L Vidal S Ramírez J Llamas-Velasco M Fernández-Carballido C The paradigm of IL-23-independent production of IL-17F and IL-17A and their role in chronic inflammatory diseases. Front Immunol 2023;14:1191782. doi: 10.3389/fimmu.2023.1191782 37600764 PMC10437113

[14] Jethwa H Abraham S. The evidence for microbiome manipulation in inflammatory arthritis. Rheumatology 2016;kew374. doi: 10.1093/rheumatology/kew374 27789760

[15] Kragsnaes MS Kjeldsen J Horn HC Munk HL Pedersen FM Holt HM Efficacy and safety of faecal microbiota transplantation in patients with psoriatic arthritis: protocol for a 6-month, double-blind, randomised, placebo-controlled trial. BMJ Open 2018;8:e019231. doi: 10.1136/bmjopen-2017-019231 PMC5922473 29703851

[16] Raimondo MG Gandolfo S Gensler LS Thomas R Schett G Ramming A From psoriatic plaque to synovium: decoding the skin–joint axis in psoriatic arthritis. Nat Rev Rheumatol 2026 Jul 31. doi: 10.1038/s41584-026-01412-3.Online ahead of print. 42538408

[17] Azuaga AB Ramírez J Cañete JD. Psoriatic Arthritis: Pathogenesis and Targeted Therapies. Int J Mol Sci 2023;24:4901. doi: 10.3390/ijms24054901 36902329 PMC10003101

[18] Cafaro G Garcia-Melchor E Akbar M Gilchrist DS Kitson SM Siebert S AB0028 Activated stromal cells induce ccl20 release and t cell migration. Ann Rheum Dis 2018;77:1216. doi: 10.1136/annrheumdis-2018-eular.4946

[19] Jacques P Lambrecht S Verheugen E Pauwels E Kollias G Armaka M Proof of concept: enthesitis and new bone formation in spondyloarthritis are driven by mechanical strain and stromal cells. Ann Rheum Dis 2014;73:437–45. doi: 10.1136/annrheumdis-2013-203643 23921997

[20] Veale DJ Fearon U. The pathogenesis of psoriatic arthritis. Lancet 2018;391:2273–84. doi: 10.1016/S0140-6736(18)30830-4 29893226

[21] Tinazzi I McGonagle D Aydin SZ Chessa D Marchetta A Macchioni P. ‘Deep Koebner’ phenomenon of the flexor tendon-associated accessory pulleys as a novel factor in tenosynovitis and dactylitis in psoriatic arthritis. Ann Rheum Dis 2018;77:922–5. doi: 10.1136/annrheumdis-2017-212681 29511028

[22] Tan AL Fukuba E Halliday NA Tanner SF Emery P McGonagle D. High-resolution MRI assessment of dactylitis in psoriatic arthritis shows flexor tendon pulley and sheath-related enthesitis. Ann Rheum Dis 2015;74:185–9. doi: 10.1136/annrheumdis-2014-205839 25261575 PMC4283670

[23] McInnes IB Kavanaugh A Gottlieb AB Puig L Rahman P Ritchlin C Efficacy and safety of ustekinumab in patients with active psoriatic arthritis: 1 year results of the phase 3, multicentre, double-blind, placebo-controlled PSUMMIT 1 trial. Lancet 2013;382:780–9. doi: 10.1016/S0140-6736(13)60594-2 23769296

[24] Ritchlin C Rahman P Kavanaugh A McInnes IB Puig L Li S Efficacy and safety of the anti-IL-12/23 p40 monoclonal antibody, ustekinumab, in patients with active psoriatic arthritis despite conventional non-biological and biological anti-tumour necrosis factor therapy: 6-month and 1-year results of the phase 3, multicentre, double-blind, placebo-controlled, randomised PSUMMIT 2 trial. Ann Rheum Dis 2014;73:990–9. doi: 10.1136/annrheumdis-2013-204655 24482301 PMC4033144

[25] McInnes IB Mease PJ Kirkham B Kavanaugh A Ritchlin CT Rahman P Secukinumab, a human anti-interleukin-17A monoclonal antibody, in patients with psoriatic arthritis (FUTURE 2): a randomised, double-blind, placebo-controlled, phase 3 trial. Lancet 2015;386:1137–46. doi: 10.1016/S0140-6736(15)61134-5 26135703

[26] Mease PJ Van Der Heijde D Ritchlin CT Okada M Cuchacovich RS Shuler CL Ixekizumab, an interleukin-17A specific monoclonal antibody, for the treatment of biologic-naive patients with active psoriatic arthritis: results from the 24-week randomised, double-blind, placebo-controlled and active (adalimumab)-controlled period of the phase III trial SPIRIT-P1. Ann Rheum Dis 2017;76:79–87. doi: 10.1136/annrheumdis-2016-209709 27553214 PMC5264219

[27] Nash P Kirkham B Okada M Rahman P Combe B Burmester GR Ixekizumab for the treatment of patients with active psoriatic arthritis and an inadequate response to tumour necrosis factor inhibitors: results from the 24-week randomised, double-blind, placebo-controlled period of the SPIRIT-P2 phase 3 trial. Lancet 2017;389:2317–27. doi: 10.1016/S0140-6736(17)31429-0 28551073

[28] Smolen JS Sebba A Ruderman EM Schulze-Koops H Sapin C Gellett AM Efficacy and Safety of Ixekizumab with or Without Methotrexate in Biologic-Naïve Patients with Psoriatic Arthritis: 52-Week Results from SPIRIT-H2H Study. Rheumatol Ther 2020;7:1021–35. doi: 10.1007/s40744-020-00250-3 33200394 PMC7695764

[29] Baraliakos X Gossec L Pournara E Jeka S Mera-Varela A D’Angelo S Secukinumab in patients with psoriatic arthritis and axial manifestations: results from the double-blind, randomised, phase 3 MAXIMISE trial. Ann Rheum Dis 2021;80:582–90. doi: 10.1136/annrheumdis-2020-218808 33334727 PMC8053347

[30] Deodhar A Helliwell PS Boehncke W-H Kollmeier AP Hsia EC Subramanian RA Guselkumab in patients with active psoriatic arthritis who were biologic-naive or had previously received TNFα inhibitor treatment (DISCOVER-1): a double-blind, randomised, placebo-controlled phase 3 trial. Lancet 2020;395:1115–25. doi: 10.1016/S0140-6736(20)30265-8 32178765

[31] Mease PJ Rahman P Gottlieb AB Kollmeier AP Hsia EC Xu XL Guselkumab in biologic-naive patients with active psoriatic arthritis (DISCOVER-2): a double-blind, randomised, placebo-controlled phase 3 trial. Lancet 2020;395:1126–36. doi: 10.1016/S0140-6736(20)30263-4 32178766

[32] Kristensen LE Keiserman M Papp K McCasland L White D Lu W Efficacy and safety of risankizumab for active psoriatic arthritis: 24-week results from the randomised, double-blind, phase 3 KEEPsAKE 1 trial. Ann Rheum Dis 2022;81:225–31. doi: 10.1136/annrheumdis-2021-221019 34911706 PMC8762015

[33] Östör A Van Den Bosch F Papp K Asnal C Blanco R Aelion J Efficacy and safety of risankizumab for active psoriatic arthritis: 24-week results from the randomised, double-blind, phase 3 KEEPsAKE 2 trial. Ann Rheum Dis 2022;81:351–8. doi: 10.1136/annrheumdis-2021-221048 34815219 PMC8862056

[34] Mease PJ Ritchlin CT Coates LC Kollmeier AP Zhou B Jiang Y Inhibition of structural damage progression with the selective interleukin-23 inhibitor guselkumab in participants with active PsA: results through week 24 of the phase 3b, randomised, double-blind, placebo-controlled APEX study. Ann Rheum Dis 2025;84:1983–94. doi: 10.1016/j.ard.2025.08.006 40962616

[35] Lonowski S Khattri S Talia J Ait-Challal B Iyile T Yamazaki H 73882 Long-Term Efficacy of Risankizumab in Maintenance of Non-Radiographic Progression in Patients With Active Psoriatic Arthritis: 5-Year Data From the KEEPsAKE 1 Phase 3 Trial. J Am Acad Dermatol 2026;95:AB288. doi: 10.1016/j.jaad.2026.04.1161

[36] McInnes IB Asahina A Coates LC Landewé R Merola JF Ritchlin CT Bimekizumab in patients with psoriatic arthritis, naive to biologic treatment: a randomised, double-blind, placebo-controlled, phase 3 trial (BE OPTIMAL). Lancet 2023;401:25–37. doi: 10.1016/S0140-6736(22)02302-9 36493791

[37] Merola JF Landewé R McInnes IB Mease PJ Ritchlin CT Tanaka Y Bimekizumab in patients with active psoriatic arthritis and previous inadequate response or intolerance to tumour necrosis factor-α inhibitors: a randomised, double-blind, placebo-controlled, phase 3 trial (BE COMPLETE). Lancet 2023;401:38–48. doi: 10.1016/S0140-6736(22)02303-0 36495881

[38] McInnes IB Gossec L Gottlieb AB Mease PJ Morita A Tanaka Y LB0001 BIMEKIZUMAB EFFICACY & SAFETY VERSUS RISANKIZUMAB IN PATIENTS WITH ACTIVE PSORIATIC ARTHRITIS: 16-WEEK RESULTS FROM A HEAD-TO-HEAD, MULTICENTRE, RANDOMISED, PHASE 3B STUDY (BE BOLD). Presented at: EULAR 2026, June 2026.

[39] Kiltz U Sfikakis PP Gaffney K Bounas A Gullick N Lespessailles E Interim 2-Year Analysis from SERENA: A Real-World Study in Patients with Psoriatic Arthritis or Ankylosing Spondylitis Treated with Secukinumab. Rheumatol Ther 2022;9:1129–42. doi: 10.1007/s40744-022-00460-x 35674938 PMC9174439

[40] Moreno-Ramos MJ Sanchez-Piedra C Martínez-González O Rodríguez-Lozano C Pérez-Garcia C Freire M Real-World Effectiveness and Treatment Retention of Secukinumab in Patients with Psoriatic Arthritis and Axial Spondyloarthritis: A Descriptive Observational Analysis of the Spanish BIOBADASER Registry. Rheumatol Ther 2022;9:1031–47. doi: 10.1007/s40744-022-00446-9 35467242 PMC9314517

[41] López-Medina C Otero-Varela L Sánchez-Alonso F Jovaní V Expósito-Pérez L Melchor-Díaz S One-year retention rate of ixekizumab in patients with psoriatic arthritis and axial spondyloarthritis: Real-world data from the BIOBADASER registry. Reumatol Clin 2025;21:501872. doi: 10.1016/j.reumae.2025.501872 40619204

[42] Mease PJ Ogdie A Tesser J Shiff NJ Lin I Chakravarty SD Six-Month Persistence and Multi-domain Effectiveness of Guselkumab in Adults with Psoriatic Arthritis: Real-World Data from the CorEvitas Psoriatic Arthritis/Spondyloarthritis Registry. Rheumatol Ther 2023;10:1479–501. doi: 10.1007/s40744-023-00582-w 37597159 PMC10654277

[43] Atzeni F Rotondo C Siragusano C Corrado A Cauli A Caporali R Italian Multicenter Real-World Study on the Twelve-Month Effectiveness, Safety, and Retention Rate of Guselkumab in Psoriatic Arthritis Patients. J Clin Med 2025;14:4111. doi: 10.3390/jcm14124111 40565857 PMC12193969

[44] Gossec L Kerschbaumer A Ferreira RJO Aletaha D Baraliakos X Bertheussen H EULAR recommendations for the management of psoriatic arthritis with pharmacological therapies: 2023 update. Ann Rheum Dis 2024;83:706–19. doi: 10.1136/ard-2024-225531 38499325 PMC11103320

[45] Coates LC Soriano ER Corp N Bertheussen H Callis Duffin K Campanholo CB Group for Research and Assessment of Psoriasis and Psoriatic Arthritis (GRAPPA): updated treatment recommendations for psoriatic arthritis 2021. Nat Rev Rheumatol 2022;18:465–79. doi: 10.1038/s41584-022-00798-0 35761070 PMC9244095

[46] Deodhar A Gensler LS Sieper J Clark M Calderon C Wang Y Three Multicenter, Randomized, Double-Blind, Placebo-Controlled Studies Evaluating the Efficacy and Safety of Ustekinumab in Axial Spondyloarthritis. Arthritis Rheumatol 2019;71:258–70. doi: 10.1002/art.40728 30225992

[47] Baeten D Østergaard M Wei JC-C Sieper J Järvinen P Tam LS Risankizumab, an IL-23 inhibitor, for ankylosing spondylitis: results of a randomised, double-blind, placebo-controlled, proof-of-concept, dose-finding phase 2 study. Ann Rheum Dis 2018;77:1295–302. doi: 10.1136/annrheumdis-2018-213328 29945918 PMC6104676

[48] Helliwell PS Gladman DD Chakravarty SD Effects of ustekinumab on spondylitis-associated endpoints in TNFinaïve active psoriatic arthritis patients with physician-reported spondylitis: pooled results from two phase 3, randomised, controlled trials. RMD Open 2020;6:e001149. doi: 10.1136/rmdopen-2019-001149 32209721 PMC7046941

[49] Mease PJ Helliwell PS Gladman DD Kafka S Karyekar CS You Y Efficacy of guselkumab on axial involvement in patients with active psoriatic arthritis and sacroiliitis: a post-hoc analysis of the phase 3 DISCOVER-1 and DISCOVER-2 studies. Lancet Rheumatol 2021;3:e715–23. doi: 10.1016/S2665-9913(21)00105-3 38287608

[50] Ruscitti P Pantano I Cataldi G Short-term effectiveness of guselkumab in psoriatic arthritis patients and axial involvement: results from a real-life multicentre cohort. Rheumatology 2025;64:1122–30. doi: 10.1093/rheumatology/keae220 38598432

[51] Tonutti A Abacar K Macleod T Gentile M Arrigoni F Riccio L Time to lift the moratorium on IL-23 inhibitors for axial psoriatic arthritis. Lancet Rheumatol 2026;8:e144–56. doi: 10.1016/S2665-9913(25)00287-5 41319674

[52] Gladman DD Mease PJ Bird P Soriano ER Chakravarty SD Shawi M Efficacy and safety of guselkumab in biologic-naïve patients with active axial psoriatic arthritis: study protocol for STAR, a phase 4, randomized, double-blinded, placebo-controlled trial. Trials 2022;23:743. doi: 10.1186/s13063-022-06589-y 36064592 PMC9444112

[53] Yamada A Wang J Komaki Y Komaki F Micic D Sakuraba A. Systematic review with meta-analysis: risk of new onset IBD with the use of anti-interleukin-17 agents. Aliment Pharmacol Ther 2019;50:373–85. doi: 10.1111/apt.15397 31309607

[54] Letarouilly J-G Pham T Pierache A Acquacalda É Banneville B Barbarot S New-onset inflammatory bowel diseases among IL-17 inhibitor-treated patients: results from the case– control MISSIL study. Rheumatology 2022;61:2848–55. doi: 10.1093/rheumatology/keab819 34730790

[55] Bissonnette R Soung J Hebert AA Pink AE Pinter A Moore AY Oral Icotrokinra for Plaque Psoriasis in Adults and Adolescents. N Engl J Med 2025;393:1784–95. doi: 10.1056/NEJMoa2504187 41191940

[56] Merola JF Mease PJ Coates L McInnes I Nash P Ogdie A 0562 Icotrokinra (ICO), a Novel Targeted Oral Peptide, in Patients (Pts) With Psoriatic Disease: Exploratory Assessments From a Phase 2 Psoriasis (PsO) Study Informing a Phase 3 Clinical Program in Psoriatic Arthritis (PsA) [abstract]. Arthritis Rheumatol 2025;77(suppl 9).

